# Vaginal Microbiota Profiles of Native Korean Women and Associations with High-Risk Pregnancy

**DOI:** 10.4014/jmb.1908.08016

**Published:** 2019-12-02

**Authors:** Dong-Ho Chang, Jongoh Shin, Moon-Soo Rhee, Kyung-Ryang Park, Byung-Kwan Cho, Sung-Ki Lee,, Byoung-Chan Kim

**Affiliations:** 1 Metabolic Regulation Research Center, Korea Research Institute of Bioscience and Biotechnology (KRIBB), Daejeon 344, Republic of Korea; 2 Department of Biological Science and Biotechnology, Hannam University, Daejeon 34054, Republic of Korea; 3 Department of Biological Sciences and KI for the BioCentury, Korea Advanced Institute of Science and Technology, Daejeon 4141, Republic of Korea; 4 Biological Resource Center, Korea Research Institute of Bioscience and Biotechnology (KRIBB), Daejeon 311, Republic of Korea; 5 Department of Obstetrics and Gynecology, College of Medicine, Myunggok Medical Research Center, Konyang University, Daejeon 336, Republic of Korea; 6 Department of Bioprocess Engineering, KRIBB School of Biotechnology, Korea University of Science and Technology (UST), Daejeon 34113, Republic of Korea

**Keywords:** Vaginal microbiota, high risk pregnancy, preterm, miscarriage

## Abstract

The vaginal microbiota may be important for pregnancy prognosis because vaginal dysbiosis during pregnancy appears to be related to preterm birth (PTB) or pregnancy loss. Previous reports have indicated that a *Lactobacillus*-poor microbial flora in the vagina and intrauterine infection by diverse anaerobes ascending from the vagina are associated with undesirable delivery outcomes. However, no research has involved the use of pyrosequencing analysis to examine vaginal microbiota profiles or their potential associations with high-risk pregnancy in Korean women. Vaginal swabs were collected from 500 Korean women for the identification of community state types (CSTs). Of these, 137 samples were further analyzed using a Roche/454 GS Junior pyrosequencer. Three distinct CSTs were identified based on the dominant vaginal microbes: CST I (*Lactobacillus crispatus* dominated), CST III (*Lactobacillus iners* dominated), and CST IV (with diverse species of anaerobes). Twelve of the 67 pregnant women had undesirable pregnancy outcomes (four miscarriages and eight PTBs). The dominant microbe in the vaginal microbiota of women who gave birth at full-term was *L. crispatus*. In contrast, *L. iners* was the dominant vaginal microbe in women who miscarried. Most (*n* = 6/8) vaginal microbiota profiles of women who experienced PTB could be classified as CST IV, with diverse bacteria, including anaerobic vaginal species. The present study provides valuable information regarding the characteristics of the vaginal microbiota of Korean women related to high-risk pregnancy. Investigation of the vaginal microbiotic structure in pregnant Korean women is necessary to enable better prediction of adverse pregnancy outcomes.

## Introduction

Since the development of pyrosequencing platform technologies, the human microbiota has received increasing attention because of its associations with aspects of human health and disease, including immune programming, protection from pathogens, and nutrient acquisition [1, 2]. In addition, the beneficial roles of the human microbiota have been investigated for application to many health issues, including obesity [3, 4], poor immune response [5], and inflammation [6]. In particular, the vaginal microbial flora reportedly plays a significant role in pregnancy, protecting the health of the mother and fetus [5, 6]. Several studies have examined the vaginal microbiota during pregnancy using culture-independent molecular techniques [7-9]. These studies have consistently shown that the vaginal microbial communities of pregnant women are dominated by *Lactobacillus* species and are characterized by less species richness and diversity, but greater stability, compared with those of non-pregnant women. A *Lactobacillus*-poor vaginal flora may disrupt the microbial balance in the vagina, often resulting in bacterial vaginosis, which is associated with high-risk pregnancy [5, 8].

Most research published to date on the human vaginal microbial ecosystem has focused on the microbiota of healthy asymptomatic women of reproductive age. The vaginal microbiota undergoes major compositional changes throughout a woman’s lifespan, from birth to puberty to menopause [10-12]. Vaginal microbial abnormality increases the risks for various obstetric and gynecological diseases and conditions, such as sexually transmitted infections [13], preterm birth (PTB) [14, 15], early and late miscarriage [16], recurrent abortion [17], histological chorioamnionitis [18] and postpartum endometritis [19]. Recently, a *Lactobacillus*-poor vaginal flora in the female reproductive tract was associated not only with high-risk pregnancy, but also with implantation failure, indicating the importance of the female microbiota for gynecological and obstetric health [20, 21].

The vaginal microbial composition may provide useful information for accurate and rapid diagnosis or prediction of pregnancy conditions. For example, Haque *et al*. insisted that vaginal microbiota enables early and highly accurate prediction of preterm delivery outcomes, and can potentially be deployed in clinical settings for preterm birth risk-assessment [22]. However, the vaginal microbiota and its association with high-risk pregnancy remain poorly understood, and in some cases, current knowledge is contradictory. Hyman *et al*. [14] reported that PTB was linked to greater intracommunity alpha-diversity in the vagina. DiGiulio *et al*. claimed that prevalence of a *Lactobacillus*-poor vaginal community state type (CST IV) was inversely correlated with gestational age at delivery [8]. In contrast, Romero *et al*. found no significant association between PTB and any specific community state type (CST) or the relative abundance of microbial phylotypes [20]. Moreover, several reports [21, 23] have indicated that the vaginal microbiota can differ based on race and ethnicity, which emphasizes the strong need for individual studies to appropriately evaluate the vaginal microbiota in various ethnic groups.

 In the present study, the vaginal microbiota profiles of 137 Korean women were examined using a 454 GS Junior pyrosequencing system (Roche). The microbial community structure and representative microbial groups in normal and high-risk pregnancy groups were identified. We then analyzed correlations between community structure and delivery outcomes, such as PTB and miscarriage, to identify specific microbial taxonomic targets for prediction. This formulation could aid the prediction of possible pregnancy outcomes and prevent reproductive health complications in Korean women.

## Methods

### Study Population and Sampling

This study received ethical approval from Konyang University Hospital Institutional Review Board (IRB) (Approval Number 2014-06-009). All participants provided written informed consent and all methods were performed in accordance with the relevant guidelines and regulations. Women attending antenatal clinics of Department of Obstetrics and Gynecology, College of Medicine, Myunggok Medical Research Center, Konyang University (Korea) between September 2014 and August 2018 were invited to be part of a clinical trial to determine the vaginal microbiome structures of Korean women. This study was conducted as a prospective observational study. For non-pregnant women, samples were obtained as being non-menstrual. The vaginal swabs were not collected at any specific non-menstrual cycle time as previous report has demonstrated there is little variation in microbiota structures through the cycle [24]. Vaginal swabs collected from pregnant women at 16-20 weeks of gestational age were used for bacterial community analysis. Vaginal swabs were collected under direct visualization using a speculum by either a physician or a nurse and placed in dry tubes prior to being placed in −80°C. A total of 137 women were enrolled in the vaginal microbiome study, including 67 pregnant women. After pyrosequencing, 11 of the 137 metagenome samples were found to have an average low read quality (Phred quality score <20), short average read length (< 250 bp) or low sequencing output (the number of reads per sample < 500). Therefore, the 11 metagenome data (7 pregnant & 4 non-pregnant) were excluded from further analysis. For the PTB group (n = 8), eligible participants for this study were women who had undergone preterm deliveries at greater than 16 weeks but less than 37 weeks, where onset of labor occurred spontaneously or in association with cervical incompetence or preterm premature rupture of membranes (PPROM). The microbial profiles of pregnant women with term-deliveries (n = 48) were compared to profiles generated from PTB (n = 8), miscarriage (n = 4) and non-pregnant Korean women (n = 66).

### PCR Amplification of 16S rRNA Genes and Pyrosequencing

Frozen vaginal swabs were sent to Chunlab, Inc. (Korea) for pyrosequencing analysis. Total nucleic acid was extracted from swabs using Mobio Soil kit (Qiagen, USA) according to the manufacturer’s instruction. PCR amplification was performed using primers targeting from V1 to V3 regions of the 16S rRNA gene with extracted DNA. For bacterial amplification, barcoded primers of 9F 5’-CCTATCCCCTGTGTGCCTTGGCAGTC-TCAG-AC-AGAGTTTGATCMTGGCTCAG-3’; underlining sequence indicates the target region primer) and 541R 5’-CCATCTCAT CCCTGCGTGTCTCCGAC-TCAG-X-AC-ATTACCGCGGCTGCTGG-3’; ‘X’ indicates the unique barcode for each subject). The amplifications were carried out under the following conditions: initial denaturation at 95°C for 5 min, followed by 30 cycles of denaturation at 95°C for 30 sec, primer annealing at 55°C for 30 sec, and extension at 72°C for 30 sec, with a final elongation at 72°C for 5 min. The PCR products were confirmed by using 2%agarose gel electrophoresis and visualized under a Gel Doc system (BioRad, USA). The amplified products were purified with the QIAquick PCR purification kit (Qiagen). Equal concentrations of purified products were pooled together and removed short fragments (non-target products) with Ampure beads kit (Agencourt Bioscience, USA). The quality and product size were assessed on a Bioanalyzer 2100 (Agilent, USA) using a DNA 7500 chip. Mixed amplicons were conducted emlusion PCR, and then deposited on Picotiter plates. The sequencing was carried out at Chunlab, Inc., with Roche/454 GS Junior Sequencing System (Roche, USA) according to the manufacturer’s instructions.

### Pyrosequencing Data Analysis

The basic analysis was conducted according to the previous descriptions in other studies [25-27]. Obtained reads from the different samples were sorted by unique barcodes of each PCR product. The sequences of the barcode, linker, and primers were removed from the original sequencing reads. Any reads containing two or more ambiguous nucleotides were discarded. Potential chimera sequences were detected by the bellerophone method, which is comparing the BLASTN search results between forward half and reverse half sequences [28]. After removing chimera sequences, the taxonomic classification of each read was assigned against the EzBioClud Database (https://www.ezbiocloud.net/ ) [29], which contains 16S rRNA gene sequence of type strains that have valid published names and representative species level phylotypes of either cultured or uncultured entries in the GenBank database with complete hierarchical taxonomic classification from the phylum to the species. The richness and diversity of samples were determined by Chao1 estimation and Shannon diversity index at the 3% distance. Random subsampling was conducted to equalize read size (n = 1,108) of samples for comparing different read sizes among samples. The overall phylogenetic distance between communities was estimated using the Fast UniFrac [30] and visualized using principal coordinate analysis (PCoA). Using CLcommunity program (Chunlab Inc.,), all the rarefaction curves were obtained (Fig. S1). To compare OTUs between samples, shared OTUs were obtained with the XOR analysis of CLcommunity program.

### Heatmap and Principal Coordinates Analysis (PCoA)

QIIME v1.9.1-dev software suite [31] was used to analyze the generated 454 pyrosequencing reads. Briefly, all reads were truncated to an even length (515 nt) using the QIIME script truncate_fasta_qual_files.py. After removal of low quality reads, operational taxonomic units (OTUs) were clustered using the QIIME script pick_open_reference_otus.py at 97% identity. An additional filtering process was conducted by first aligning all OTU sequences to Greengenes 13_8 Database using PYNAST version 1.2.2 [32]. OTU taxonomy was determined using Ribosomal Database Project classifier. Principle Coordinate Analysis (PCoA) was performed by calculating weighted and unweighted UniFrac distance between each pair of samples (QIIME script function beta_diversity_through_plots.py) on a normalized OTU table.

### Data Availability

Raw sequence data files for the 126 samples described in this study are available in the European Nucleotide Archive under study accession PRJEB33541. Due to ethical and legal restrictions related to protecting participant privacy imposed by Konyang Medical School IRB, all other relevant data are available upon request pending ethical approval.

## Results

### Sample Collection and Pregnancy Outcomes

In the present study, we characterized the vaginal microbiota profiles of pregnant and non-pregnant native Korean women. We collected vaginal swabs at 16–20 weeks of gestation. The possibility of PTB or miscarriage was usually assessed during the first or second trimester, and therapeutic interventions at this gestational stage have been considered to be efficacious [33]. In addition, pregnant women in Korea first visit the hospital at this gestational age, at which time vaginal swabs are taken to screen for vaginal infection.

From September 2014 to August 2018, we collected more than 500 vaginal swabs from native Korean women (430 pregnant and 70 non-pregnant). A portion of the collected vaginal swabs (137 samples collected from September 2014 to October 2016) was sent to Chunlab, Inc. for next-generation sequencing (NGS) analysis. Initially, Chunlab, Inc. used a GS Junior sequencing system (Roche) for the metagenome analysis of 16S rRNA gene amplicons; due to the unavailability of the Roche 454 platform service, the Illumina MiSeq sequencing system (Illumina, USA) service has been used since January 2017. Therefore, our reporting on the vaginal microbiota profiles of pregnant and non-pregnant Korean women and their possible associations with undesirable delivery outcomes is based on the 137 initially collected vaginal swab samples [from 67 pregnant women (with 55 term deliveries, 8 preterm deliveries, and 4 miscarriages) and 70 non-pregnant women] analyzed using the Roche 454 NGS platform. The collection of vaginal samples is ongoing, and when the additional 30+ samples from women who experienced PTB are analyzed using the MiSeq platform, the updated results will be reported in combination with the present work. To date, 20 additional swabs from women who experienced PTB and 6 additional swabs from women who miscarried have been collected and are being analyzed using the Illumina MiSeq pyrosequencer. Currently, the total numbers of samples from women with PTB and miscarriage outcomes are 28 and 10, respectively.

Among the 137 samples submitted for NGS using the Roche 454 platform, 11 metagenome sequences (from 7 pregnant and 4 non-pregnant women) were of insufficient quality for further analyses, such as heatmap analysis and principal coordinates analysis (PCoA). Therefore, NGS data from only 126 samples [from 60 pregnant women (with 48 term births, 8 PTBs, and 4 miscarriages) and 66 non-pregnant women] were used; corresponding personal information is summarized in Table 1. The sociodemographic characteristics of 48 women who gave birth at term, 28 women who had preterm deliveries (8 samples analyzed with the Roche 454 and 20 analyzed with the Illumina MiSeq platform), and 10 women who miscarried (4 samples analyzed with the Roche 454 and 6 analyzed with the Illumina MiSeq platform) are summarized in Table 2 and in Supplemental Table 1, respectively.

### Operational Taxonomic Unit Analysis and Microbiota Profiles

**Fig. 1:**
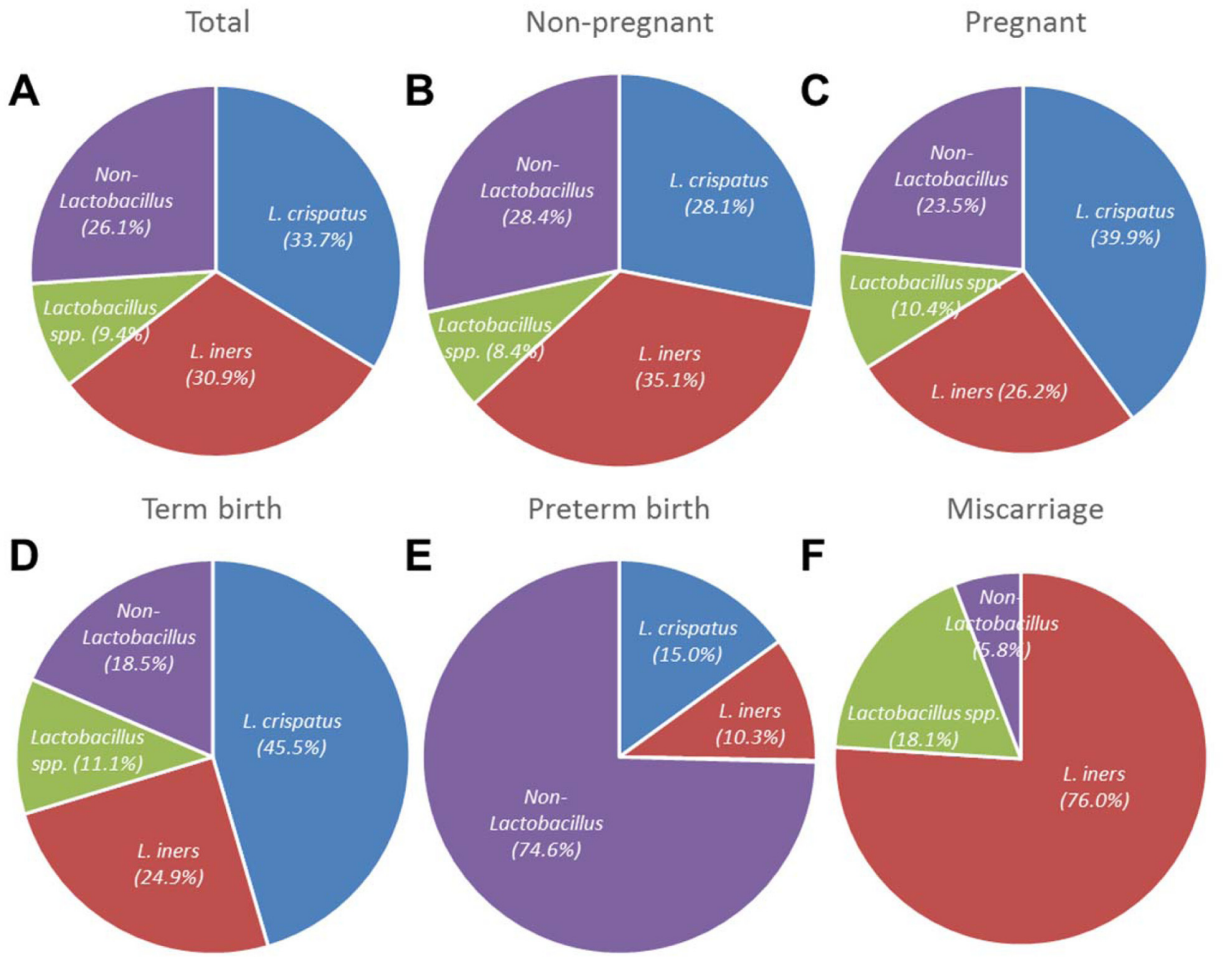
Proportion of vaginal microbes in non-pregnant and pregnant women in term, preterm and miscarriage groups based on Roche/454 pyrosequencing analysis.

The Roche 454 NGS raw sequence data files for the 126 samples described in this study are available in the European Nucleotide Archive under study accession number PRJEB33541. In total, 1,068,077 16S rRNA gene reads were generated. The median and average read counts per sample were 4,021 and 6,698 (range, 398–42,166), respectively. The average read length was 431 bp. In total, 352 families, 235 genera, and 64 bacterial species were identified. Most (73.9%) reads were identified as *Lactobacillus* spp. The two most abundant OTUs, which corresponded to *Lactobacillus* species, accounted for 64.6%of all reads generated (33.7% *Lactobacillus crispatus* and 30.9% *Lactobacillus iners*). Additional *Lactobacillus* OTUs were found, accounting for 9.4% of all reads. The remainder of the vaginal microbes (non-*Lactobacillus*) generally belonged to strictly anaerobic bacteria (26.1%; Fig. 1). Species with the most read numbers were *L. crispatus* [358,089 (33.7%)], *L. iners* [327,884 (30.9%)], *Lactobacillus jensenii* [38,601 (3.6%)], and *Atopobium vaginae* [35,148 (3.3%)]. Species with the greatest sample prevalence were *L. iners* [84/126 (66.7%)], *L. crispatus* [82/126 (65.1%)], *Lactobacillus helveticus* [46/126 (36.5%)], and *Ureaplasma parvum* [38/126 (30.2%)]. Although reads with similarity to *L. helveticus* were detected in more than 36% (46/126) of samples, they were in very low abundance, representing only 0.02% of all reads. Overall, most species with the most abundant reads and greatest prevalence were of the genus *Lactobacillus* (Table 3).

### Abundance of *Lactobacillus* spp.

The proportion of reads from *Lactobacillus* spp. in all 126 vaginal samples was 73.9% (785,508/1,062,319; Fig. 1A). The microbiota profiles of pregnant women (including women in the PTB and miscarriage groups) were compared with those of non-pregnant women. In the pregnant cohort, the proportion of *L. crispatus* (39.9%) was larger than that of *L. iners* (28.1%). In addition, the proportion of the non-*Lactobacillus* spp. was smaller in pregnant women (23.5%) than in non-pregnant women (28.4%; Figs. 1B and 1C). In particular, the proportion of *L. crispatus* from women with term deliveries (45.5%) was much larger than the proportions from pregnant (39.9%) and non-pregnant (28.1%) women (Fig. 1D). In contrast, the proportion of non-*Lactobacillus* spp. was 74.6% for women with preterm deliveries (Fig. 1E). Interestingly, *L. iners* was the most abundant bacterial species in the miscarriage group (Fig. 1F). Similar to previous studies, the proportion of *Lactobacillus* spp. from vaginal swabs followed a bimodal distribution in the present study (Fig. S2). The microbiota of most non-pregnant women had low (0–20%; *n* = 19/66) or high (80–100%; *n* = 43/66) abundances of *Lactobacillus* spp.; only 4/66 (6.1%) swabs exhibited intermediate (20–80%) levels. For pregnant women with term deliveries, 10/ 48 swabs had low (0–20%) and 34/48 swabs had high (80–100%) abundances of *Lactobacillus* spp. Only 4/48 (8.3%) swabs had intermediate (20–80%) levels. No such bimodal distribution was observed for the PTB or miscarriage group. Most [6/8 (75%)] swabs from the PTB group had low (0–20%) abundances, and all [4/4 (100%)] swabs from the miscarriage group had high (80–100%) abundances of *Lactobacillus* spp. (Fig. S2).

### Community State Type Analysis

**Fig. 2:**
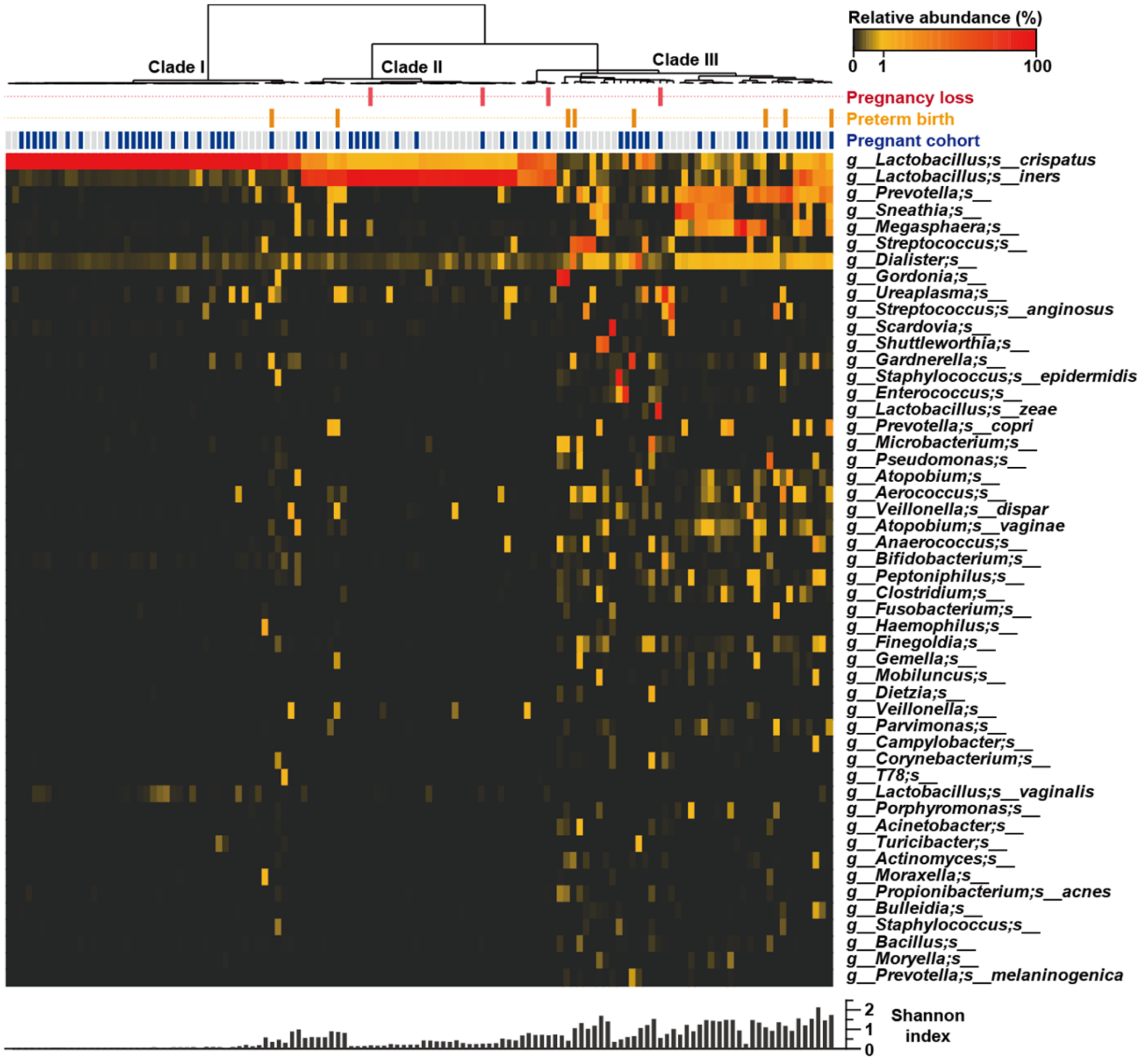
Heatmap of bacterial relative abundance by individual vaginal samples. Each column represents the relative bacterial abundance of an individual vaginal sample with the 50 most abundant species showed with their taxonomies. The dendrogram was drawn based on the hierarchical clustering solution (Ward’s method) of the 126 vaginal microbiome samples. Shannon diversity indices calculated from each vaginal samples.

Ravel and Gajer [34] previously clustered vaginal microbial communities into five groups: four were dominated by *Lactobacillus crispatus* (CST I), *L. gasseri* (CST II), *L. iners* (CST III), or *L. jensenii* (CST V), whereas the fifth had lower proportions of lactic acid bacteria and higher proportions of strictly anaerobic organisms (CST IV). In the present study, hierarchical clustering of vaginal microbiota profiles from native Korean pregnant and non-pregnant women resulted in the resolution of three clearly distinct CSTs (Fig. 2). Only two *Lactobacillus*-dominated CSTs [CST I and CST III], based on pyrosequencing of the 16 S rRNA gene, were detected frequently. Most (84/126) profiles were dominated by one of two *Lactobacillus* species among three CSTs: CST I (*L. crispatus*; *n* = 45), CST III (*L. iners*; *n* = 33) and CST I + III (an approximately equal mixture of *L. crispatus* and *L. iners*; *n* = 6). All (*n* = 42) non-*Lactobacillus*–dominated samples were assigned to CST IV, the most heterogeneous group, which included mixtures of genera such as *Atopobium* and *Gardnerella* and combinations of *Prevotella*, *Sneathia*, *Megasphaera*, *Streptococcus*, *Dialister*, and *Ureaplasma*.

**Fig. 3:**
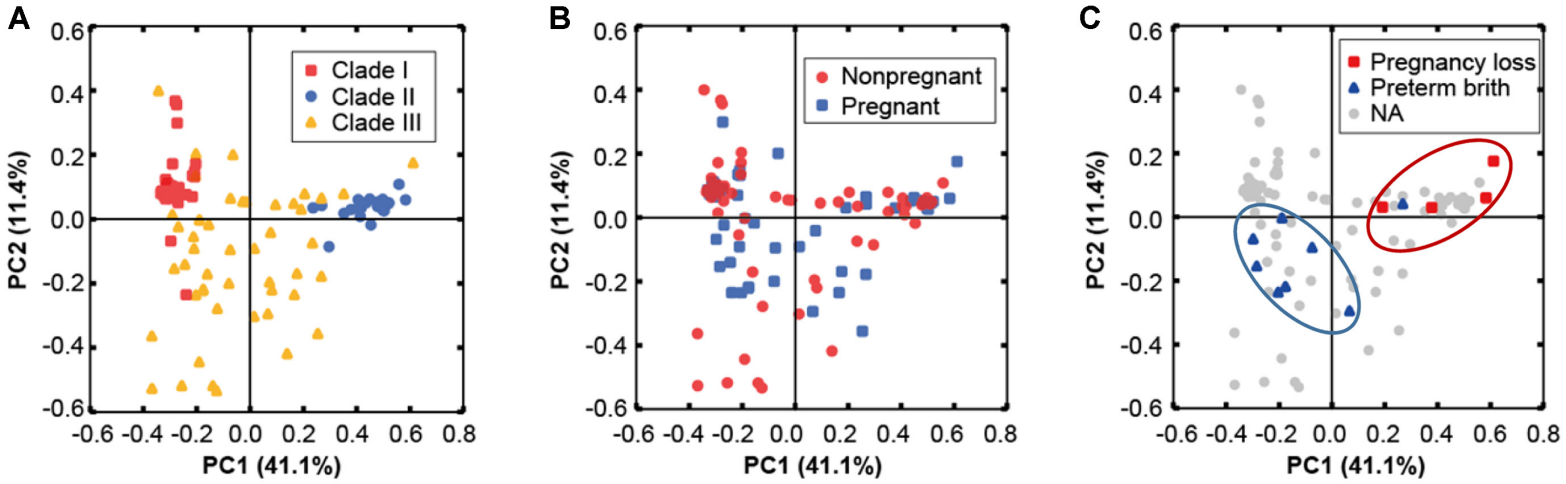
Principal Coordinates Analysis (PCoA) of weighted UniFrac distances of microbial profiles from all participants. Samples are colored by clade types (**A**), pregnancy status (**B**), and miscarriage/preterm delivery cases (**C**).

The CSTs of pregnant women in the term delivery group were compared with those in the non-pregnant group. In the latter group, 26/66 (39.4%) samples were assigned to CST IV, with the remainder assigned to the *Lactobacillus*-dominated CST I [20/66 (30.3%)] and CST II I [20/66 (30.3%)]. In the pregnant group with term deliveries, 34/48 samples were assigned to CST I [24/48 (50.0%)] and CST III [10/48 (20.8%)], with the remainder [14/48 (29.2%)] assigned to the non-*Lactobacillus*–dominated CST IV. In the PCoA ordination, the overall microbial CST could not be differentiated based on pregnancy status (Fig. 3A). Most [6/8 (75%)] samples from the PTB group were assigned to CST IV, with the remainder assigned to CST I [1/8 (12.5%)] and CST III [1/8 (12.5%)]. In contrast, most [3/4 (75%)] samples from the miscarriage group were assigned to CST III, with the remainder [1/4 (25%)] assigned to CST IV.

### Alpha Diversity Analysis

The assessment of alpha diversity revealed that the microbiomes of pregnant women with term deliveries (*n* = 48) were slightly less diverse (Shannon diversity index, 2.2± 0.1) and less rich (Chao1, 85.1 ± 9.8) compared with those of non-pregnant women (2.7 ± 0.1 and 134 ± 24.3, respectively; Fig. S3).

### Prevalence and Abundance of *Ureaplasma parvum*

*Ureaplasma* spp. are Gram negative bacteria which frequently colonize the genitourinary tract [35]. There is supporting evidence that *Ureaplasma* spp. may act as low grade pathogens in pregnancy [36]. Recently, Cox *et al*. reported that the common vaginal commensal bacterium *Ureaplasma parvum* is associated with chorioamnionitis in extreme preterm labor [37]. Roche 454 pyrosequencing detected reads of Mollicutes (*Ureaplasma* and/or *Mycoplasma*) in 30/66 (45.5%) samples from non-pregnant women, 25/60 (41.7%) samples from pregnant women in the term delivery group, 3/8 (37.5%) samples from women with preterm deliveries, and 1/4 (25%) samples from women who miscarried, but the prevalence varied among CSTs: 28.9%, 48.5%, and 19.0% in CSTs I, III, and IV, respectively. *U. parvum* was detected in samples from 18/ 66 (27.3%) non-pregnant women, 16/48 (33.3%) pregnant women with term deliveries, 3/8 (37.5%) women with preterm deliveries, and 1/4 (25%) women who miscarried. The prevalence of *U. parvum* did not vary significantly according to pregnancy status or delivery outcome. However, *U. parvum* was much less abundant in vaginal swabs from pregnant women in the term delivery group (0.08%) than in those from the non-pregnant (3.13%), PTB (0.93%), and miscarriage (1.71%) groups.

## Discussion

The NGS data for the vaginal microbiota profiles of 126 Korean women are summarized in Fig. 1. The microbial profiles from pregnant women clustered into three CSTs (I, III, and IV, originally defined by Ravel and Gajer [34]); CST II (*L. jensenii* dominant) and CST V (*L. gasseri* dominant) were not detected in the 126 samples analyzed by Roche 454 sequencing in this study (Fig. 2). Among the pregnant women, 48/60 (80.0%) delivered at >36^+6^ gestational weeks (mean, 39^+1^ weeks), and 8/60 (13.3%) delivered at <37^+0^ gestational weeks (mean, 34^+1^ weeks). The remaining four (6.7%) women miscarried at gestational ages > 18 weeks. The mean birth weights of newborns in the term delivery and PTB groups were 3,366 ± 72 and 2,260 ± 140 g, respectively. Based on the major vaginal microbial taxa (>1% of total reads in NGS analysis), single *Lactobacillus* species were detected in 64.6% of samples from women with term deliveries and 87.5% of those from women with preterm deliveries. Interestingly, the microbiota of three (75%) of the four women in the miscarriage group were dominated by *L. iners*; that of the fourth (25%) woman was dominated by *U. parvum* (12.6%, based on read numbers). Using more than 785,508 *Lactobacillus*-specific pyrosequencing reads belonging to 42 species, we verified that *Lactobacillus* dominated the vaginal microbiota of pregnant women. More than 505,819 *Lactobacillus*-specific reads belonging to 37 species were detected in vaginal swabs from women who delivered at term. In contrast, only six *Lactobacillus* species were detected in pyrosequencing reads from women who had PTBs. The *Lactobacillus* species detected in women who delivered at term were primarily *L. crispatus* (45.5%) and *L. iners* (24.9%), followed by *L. jensenii* (4.8%). Furthermore, *L. delbrueckii*, *L. acidophilus*, *L. psittaci*, *L. pontis*, *L. vaginalis*, and *L. helveticus* were detected only in several women in the term delivery group. Non-*Lactobacillus* species (74.6%) were dominant in the vaginal swabs from women who had PTBs; *A. vaginae* (17.2%), *Prevotella amnii* (9.2%), *Streptococcus agalactiae* (9.1%), *G. vaginalis* (8.5%), *Megasphaera* sp. (8.2%), and *Escherichia coli* (2.6%) were most common. Most (6/8) vaginal microbiota profiles from the PTB group could not be classified as *Lactobacillus* dominant, and were assigned to the diverse CST IV. Considering both read numbers and the prevalence of vaginal microbes, 75% (6/8) and 62.5% (5/8) of vaginal swabs from the PTB group contained reads specific to *G. vaginalis* and *A. vaginae*, respectively. These results suggest that *G. vaginalis* and *A. vaginae* may serve as taxonomic biomarkers of high-risk pregnancy and the risk of PTB, consistent with previous reports [38, 39].

Overall, the microbiota profiles of pregnant women could not be distinguished from those of non-pregnant women. However, several differences were observed between the microbiota profiles of pregnant women with term deliveries and those of non-pregnant women. Pregnant women with term deliveries exhibited much greater relative abundances of *Lactobacillus* spp. (*p value*: 0.0341) compared with non-pregnant women (Fig. 1). The vaginal microbiota of pregnant women had less diversity, richness, and less abundance of *U. parvum* than did that of non-pregnant women, consistent with previous report [40]. However, the prevalence of *U. parvum* was not different between non-pregnant women (33.3%) and pregnant women with term deliveries (37.5%), which is not consistent with the previous report [41].

In the present study, *L crispatus* and *L. iners* were identified as dominant microbial species in the vaginal microbial flora of 126 Korean women. In addition, women with normal pregnancies exhibited less bacterial diversity and greater abundances of *Lactobacillus* species compared with non-pregnant women. These results are quite consistent with those of previous studies [40, 42]. In the vagina, *Lactobacillus* spp. generate a low-pH environment through lactic acid production, which inhibits vaginal pathogens. Despite the observed variation in the microbial composition of samples from women who experienced PTB, we detected clear decreases in populations of *Lactobacillus* spp. and concomitant increases in a diverse non-*Lactobacillus* bacterial flora. *A. vaginae*, *Streptococcus* sp., *Prevotella* sp., *L. amnionii*, *Ureaplasma* sp., and *G. vaginalis* were the main non-*Lactobacillus* species identified in samples from the PTB group. These bacterial species are presumed to be the causative vaginal microbes in vaginitis [43, 44]. Therefore, improvement of the vaginal environment with antibacterial and lactic acid–generating *Lactobacillus* spp., such as *L. crispatus*, may help to prevent PTB. In the PCoA, the cases of PTB (*n* = 8) and miscarriage (*n* = 4) clustered distinctly in the ordination space and therefore had similar vaginal microbial profiles (Fig. 3C). Based on these correlations, high-risk pregnancy outcomes, such as preterm delivery and miscarriage, may be predictable. In conclusion, we identified several differences in the vaginal microbiota profiles of native Korean pregnant and non-pregnant women. Less diversity, greater abundance of *Lactobacillus*, and less abundance but no difference of prevalence of *U. parvum* were observed in Korean pregnant women in the term delivery group. These findings represent an important step for exploitation of the diagnostic potential of microbiota profiles for the prediction of high-risk pregnancy in Korea, as well as for the development of alternative therapeutic treatments involving microbiological intervention.

## Supplemental Materials



Supplementary data for this paper are available on-line only at http://jmb.or.kr.

## Figures and Tables

**Table 1:** Socio-demographic characteristics of non-pregnant and pregnant women.

Characteristics	Non-pregnant (*n*=66)	pregnant (*n*=60)	*p* value
Age(Mean SD,Range)	38±1.1 (20-58)	33.2±0.42 (26-40)	<0.0001
20-30	13	13	
31-35	20	32	
36-40	10	15	
41-50	16	-	
51-60	7	-	
Missing data	-	-	
Height(Mean SD,Range)	160.1±0.5 (149-174)	162.6±0.6 (153-174)	0.002
146-150	2	-	
151-155	8	3	
156-160	27	20	
161-165	21	24	
166-170	3	8	
171-175	2	2	
Missing data	3	3	
Weight(Mean SD,Range)	58.2±1.1 (51-98)	64.4±1.4 (48.4-97)	0.001
40-50	8	2	
51-60	39	21	
61-70	13	23	
71-80	2	7	
81-90	-	1	
91-100	1	3	
Missing data	3	3	
BMI(Mean SD,Range)	22.8±0.4 (15.82-38.3)	24.2±0.5 (17.1-36.48)	0.031
Underweight(<18.50)	3	3	
Normal weight(18.51<24.9)	48	30	
Overweight(25.0-29.9)	9	20	
Obese (>30)	3	4	
Missing data	3	3	

**Table 2:** Socio-demographic characteristics of pregnant women in term and preterm groups.

Characteristics	Term birth ≥37 weeks (*n*=48)	Preterm birth <37 week (*n*=28)	*p* value
Age(Mean SD,Range)	33±05 (26-40)	33.7±0.7 (26-41)	0.503
20-30	11 (22.9%)	6 (21.4%)	
31-35	25 (52.1%)	12 (42.9%)	
36-40	12 (25.0%)	9 (32.1%)	
41-50	-	1 (3.6%)	
Missing data	-	-	
Height(Mean SD,Range)	163.4±0.6 (153-174)	159.5±0.7 (153-166.4)	<0.0001
145-150	-	-	
151-155	3 (6.3%)	5 (17.9%)	
156-160	10 (20.8%)	15 (53.6%)	
161-165	22 (45.8%)	6 (21.4%)	
166-170	8 (16.7%)	2 (7.1%)	
171-175	2 (4.2%)	-	
Missing data	3 (6.3%)	-	
Weight(Mean SD,Range)	65.1±1.7 (62-97)	63.9±1.4 (49.8-86)	0.615
40-50	2 (4.2%)	1 (3.6%)	
51-60	16 (33.3%)	7 (25.0%)	
61-70	16 (33.3%)	18 (64.3%)	
71-80	7 (14.6%)	2 (7.1%)	
81-90	1 (2.1%)	-	
91-100	3 (6.3%)	-	
Missing data	3 (6.3%)	-	
BMI(Mean SD,Range)	24.4±0.6 (17.1-36.48)	24.9±0.6 (19.7-31.9)	0.543
Underweight(<18.50)	3 (6.3%)	-	
Normal weight(18.51<24.9)	23 (47.9%)	13 (46.4%)	
Overweight(25.0-29.9)	15 (31.3%)	13 (46.4%)	
Obese (>30)	4 (8.3%)	2 (7.1%)	
Missing data	3 (6.3%)	-	
Delivery			0.572
Natural			
childbirth	19 (39.6%)	11 (39.3%)	
Cesarean	16 (33.3%)	17 (60.7%)	
Missing data	13 (27.1%)	-	
Pregnancy			0.902
Naturally conceived	35 (72.9%)	24 (85.7%)	
Embryo transfer	1 (2.1%)	4 (14.3%)	
Unknown	12 (25.0%)	-	
Gestational weeks at delivery	39^+1^ (37^+1^-41^+2^)	34^+1^ (17-36^+6^)	<0.0001
delivery of times			0.620
0	10 (20.8%)	12 (42.9%)	
1	9 (18.8%)	7 (25%)	
>2	24 (50%)	9 (32.1%)	
missing data	5 (10.4%)	-	
Baby			
Boy	14	16	
Girl	21	15	
Missing data	13	-	
Weight(g)	3366±72	2385±144	<0.0001
Weight(<2500g)	1	17	

**Table 3:** Prevalence and proportion of total reads for “species” detected in at least 10% of samples.

Species	Prevalence/126(%)	% total reads	Reads
*Lactobacillus* sp.	93 (73.8%)	0.3	3,470
*Lactobacillus iners*	84 (66.7%)	30.9	327,884
*Lactobacillus crispatus*	82 (65.1%)	33.7	358,089
*Lactobacillaceae*	80 (63.5%)	0.0	444
*Lactobacillus helveticus*	46 (36.5%)	0.0	260
*Lactobacillus* sp.	42 (33.3%)	0.2	2,514
*Ureaplasma parvum*	38 (30.2%)	1.8	18,741
*Atopobium vaginae*	36 (28.6%)	3.3	35,148
*Lactobacillus psittaci*	36 (28.6%)	0.5	5,604
*Lactobacillus jensenii*	35 (27.8%)	3.6	38,601
*Gardnerella vaginalis*	31 (24.6%)	0.4	4,761
*Prevotella timonensis*	31 (24.6%)	0.7	7,661
*Dialister micraerophilus*	31 (24.6%)	0.5	5,368
*Prevotella bivia*	28 (22.2%)	1.4	15,402
*Prevotella* sp.	27 (21.4%)	0.0	344
*Lactobacillus vaginalis*	26 (20.6%)	0.0	356
*Megasphaera* sp.	24 (19.0%)	2.0	20,893
*Lactobacillus kitasatonis*	24 (19.0%)	0.0	85
*Lactobacillus ultunensis*	23 (18.3%)	0.0	85
*Veillonellaceae*	22 (17.5%)	0.0	72
*Dialister* sp.	21 (16.7%)	0.2	2,311
*Aerococcus christensenii*	21 (16.7%)	0.1	1,053
*Prevotellaceae*	21 (16.7%)	0.0	154
*Atopobium* sp.	21 (16.7%)	0.0	72
*Megasphaera* sp.	20 (15.9%)	0.0	133
*Lactobacillus gasseri*	18 (14.3%)	2.2	23,384
*Peptoniphilus indolicus*	18 (14.3%)	0.0	326
*Sneathia sanguinegens*	17 (13.5%)	0.7	7,535
*Coriobacteriaceae*	17 (13.5%)	0.2	1,745
*Microbacterium laevaniformans group*	17 (13.5%)	0.1	705
*Leptotrichia amnionii*	16 (12.7%)	2.8	29,310
*Lactobacillus rodentium*	16 (12.7%)	0.0	254
*Lactobacillus* sp.	16 (12.7%)	0.0	61
*Lactobacillales*	16 (12.7%)	0.0	20
*Streptococcus anginosus*	15 (11.9%)	1.1	12,176
*Dialister* sp.	15 (11.9%)	0.0	48
*Gordonia sputi group*	14 (11.1%)	0.4	4,004
*Lactobacillus* sp.	14 (11.1%)	0.1	649
*Propionibacterium acnes*	14 (11.1%)	0.0	50
*Ruminococcaceae*	13 (10.3%)	0.2	1,672
*Prevotella* sp.	13 (10.3%)	0.0	480
*Dialister propionicifaciens*	13 (10.3%)	0.0	73
The Remainder species		12.7	136,080
