# Supplementary material for: Vaginal Microbiota Profiles of Native Korean Women and Associations with High-Risk Pregnancy
Source: J Microbiol Biotechnol. 2019 Dec 2;30(2):248–58. doi: 10.4014/jmb.1908.08016 (PMC9728229; doi:10.4014/jmb.1908.08016)

J. Microbiol. Biotechnol.
https://doi.org/10.4014/jmb.1908.08016

Supplemental Data

Supplemental Table 1 Socio-demographic characteristics of pregnant women in term and miscarriage groups

Characteristics	Term birth >37 weeks (n=48)	Miscarriage (n=10)	*p* value
Age(Mean SD,Range)	33±05 (26-40)	33.3±0.96 (30-38)	0.876
20-30	11 (22.9%)	2 (20%)	
31-35	25 (52.1%)	6 (60%)	
36-40	12 (25.0%)	2 (20%)	
41-50	-	-	
Missing data	-	-	
Height(Mean SD,Range)	163.4±0.6 (153-174)	161±1.8 (153-172)	0.1457
145-150	-	-	
151-155	3 (6.3%)	1 (10%)	
156-160	10 (20.8%)	6 (60%)	
161-165	22 (45.8%)	-	
166-170	8 (16.7%)	2 (20%)	
171-175	2 (4.2%)	1 (10%)	
Missing data	3 (6.3%)	-	
Weight(Mean SD,Range)	65.1±1.7 (62-97)	59.68±2.2 (51-74)	0.150
40-50	2 (4.2%)	-	
51-60	16 (33.3%)	6 (60%)	
61-70	16 (33.3%)	3 (30%)	
71-80	7 (14.6%)	1 (10%)	
81-90	1 (2.1%)	-	
91-100	3 (6.3%)	-	
Missing data	3 (6.3%)	-	
BMI(Mean SD,Range)	24.4±0.6 (17.1-36.48)	22.72±0.92 (19.6-27.3)	0.221
Underweight(<18.50)	3 (6.3%)	-	
Normal weight(18.51<24.9)	23 (47.9%)	7 (70%)	
Overweight(25.0-29.9)	15 (31.3%)	3 (30%)	
Obese (>30)	4 (8.3%)		
Missing data	3 (6.3%)	-	
Delivery			0.019
Natural childbirth	19 (39.6%)	4 (40%)	

Cesarean	16 (33.3%)	-	
Missing data	13 (27.1%)	6 (60%)	
Pregnancy			0.286
Naturally conceived	35 (72.9%)	1 (10%)	
Embryo transfer	1 (2.1%)	1 (10%)	
Unknown	12 (25.0%)	8 (80%)	
Gestational weeks at delivery	39^{+1} (37^{+1}-41^{+2})	10^{+3} (6-23^{+6})	<0.0001
delivery of times			0.358
0	10 (20.8%)	-	
1	9 (18.8%)	1 (10%)	
>2	24 (50%)	9 (90%)	
missing data	5 (10.4%)	-	

Supplemental Figure 1 rarefaction curves showing number of OTUs from 126 vaginal samples

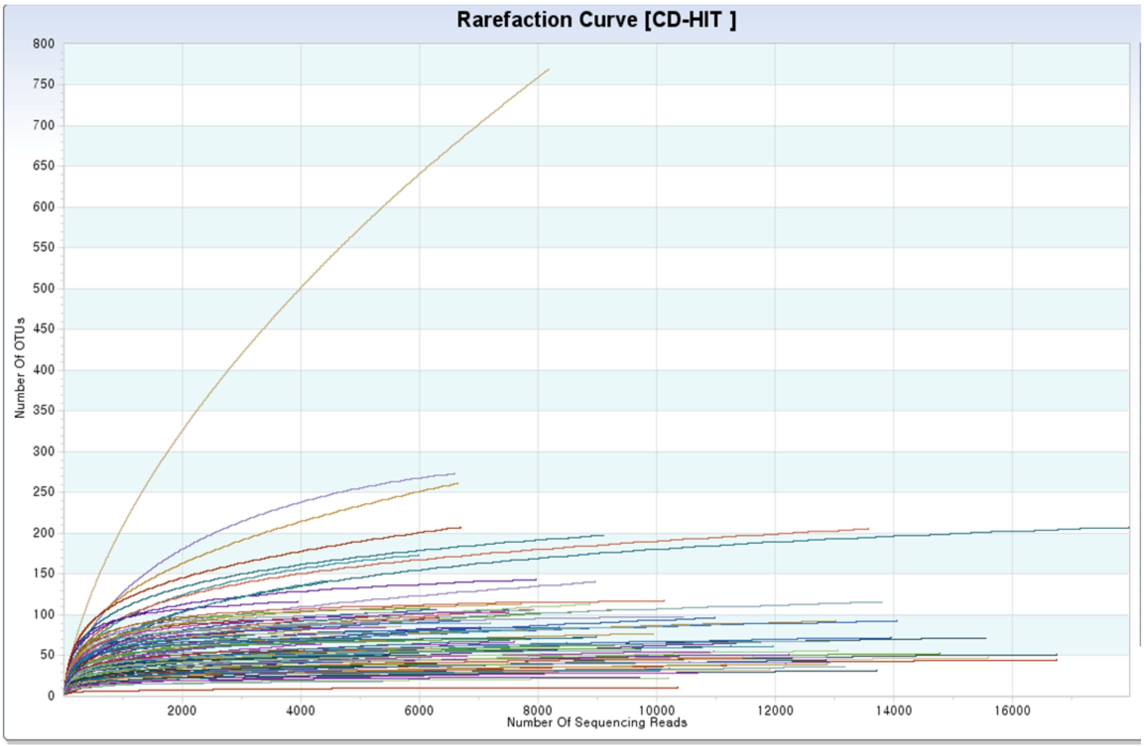

Supplemental Figure 2 *Lactobacillus* sp. abundance (%). Bimodal distribution of vaginal microbiota profiles based on pregnancy status.

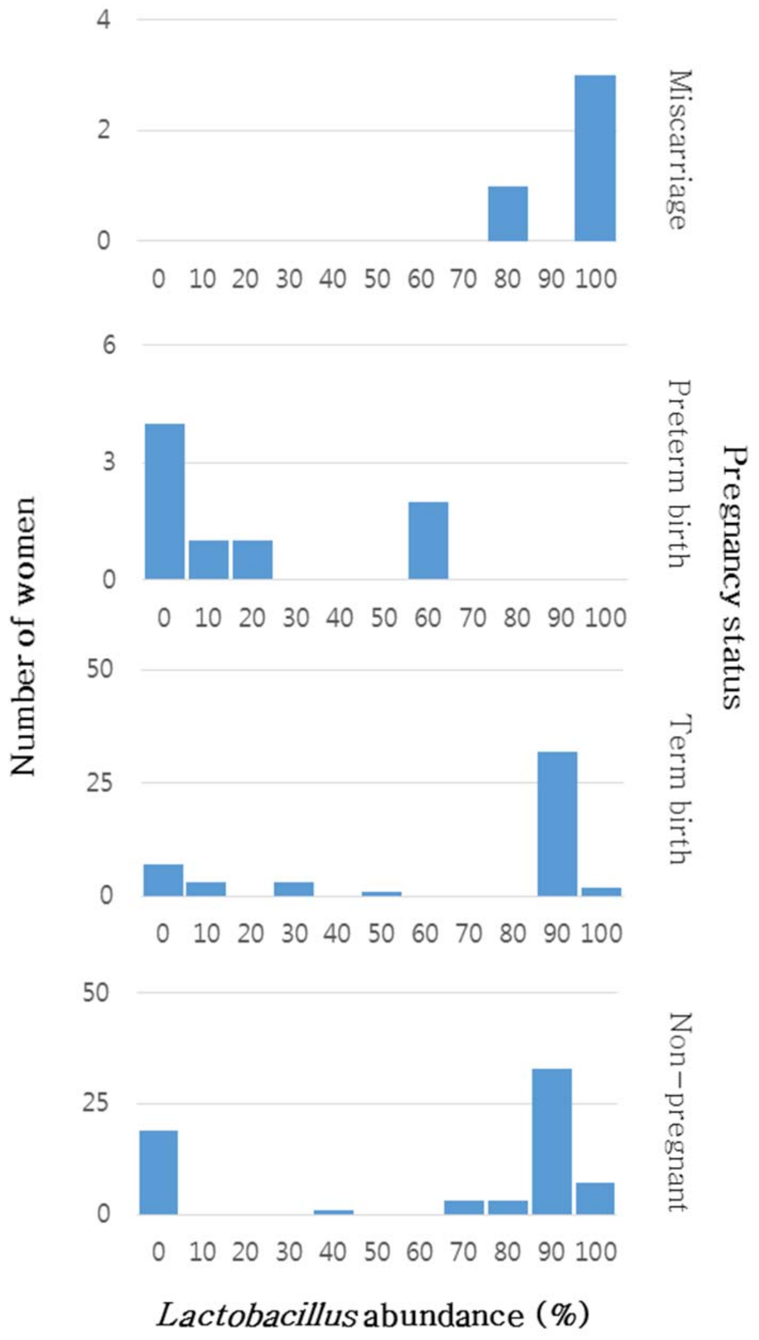

Supplemental Figure 3. Comparison of the microbial community features. *Lactobacillus* sp. abundance (A), Chao1 richness (B), Shannon diversity (C) were compared between pregnant and non-pregnant women based on pregnancy status.

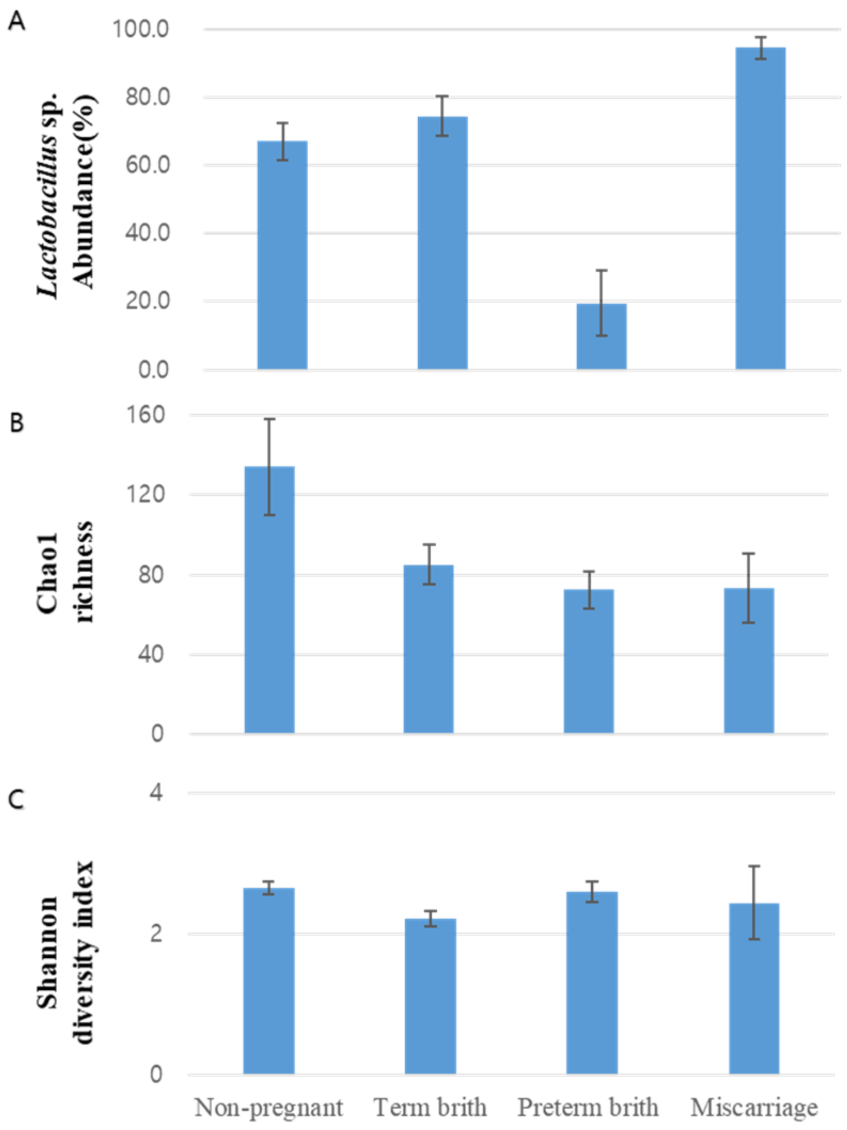

Supplement: Supplementary file 1 [file JMB-30-2-248-supple.pdf]
